# Myeloperoxidase-oxidized high density lipoprotein impairs atherosclerotic plaque stability by inhibiting smooth muscle cell migration

**DOI:** 10.1186/s12944-016-0388-z

**Published:** 2017-01-10

**Authors:** Boda Zhou, Lingyun Zu, Yong Chen, Xilong Zheng, Yuhui Wang, Bing Pan, Min Dong, Enchen Zhou, Mingming Zhao, Youyi Zhang, Lemin Zheng, Wei Gao

**Affiliations:** 1 Department of Cardiology, Key Laboratory of Molecular Cardiovascular Sciences of Ministry of Education, and Key Laboratory of Cardiovascular Molecular Biology and Regulatory Peptides of Ministry of Health, Peking University Third Hospital, No. 49 North Garden Road, Haidian District, Beijing, 100191 China; 2 The Institute of Cardiovascular Sciences and Institute of Systems Biomedicine, School of Basic Medical Sciences, Peking University Health Science Center, 38 Xueyuan Road, Haidian District, Beijing, 100191 China; 3 Department Biochemistry & Molecular Biology, the University of Calgary, Alberta, Canada; 4 Department of Neurology, People’s Hospital of Deyang City, Deyang, 618000 China

**Keywords:** HDL, Smooth muscle cell, Atherosclerosis, Protein kinases/MAP kinase, MPO, Migration, Proliferation

## Abstract

**Background:**

High density lipoprotein (HDL) has been proved to be a protective factor for coronary heart disease. Notably, HDL in atherosclerotic plaques can be nitrated (NO_2_-oxHDL) and chlorinated (Cl-oxHDL) by myeloperoxidase (MPO), likely compromising its cardiovascular protective effects.

**Method:**

Here we determined the effects of NO_2_-oxHDL and Cl-oxHDL on SMC migration using wound healing and transwell assays, proliferation using MTT and BrdU assays, and apoptosis using Annexin-V assay in vitro, as well as on atherosclerotic plaque stability in vivo using a coratid artery collar implantation mice model.

**Results:**

Our results showed that native HDL promoted SMC proliferation and migration, whereas NO_2_-oxHDL and Cl-oxHDL inhibited SMC migration and reduced capacity of stimulating SMC proliferation as well as migration, respectively. OxHDL had no significant influence on SMC apoptosis. In addition, we found that ERK1/2-phosphorylation was significantly lower when SMCs were incubated with NO_2_-oxHDL and Cl-oxHDL. Furthermore, transwell experiments showed that differences between native HDL, NO_2_-oxHDL and Cl-oxHDL was abolished after PD98059 (MAPK kinase inhibitor) treatment. In aortic SMCs from scavenger receptor BI (SR-BI) deficient mice, differences between migration of native HDL, NO_2_-oxHDL and Cl-oxHDL treated SMCs vanished, indicating SR-BI’s possible role in HDL-associated SMC migration. Importantly, NO_2_-oxHDL and Cl-oxHDL induced neointima formation and reduced SMC positive staining cells in atherosclerotic plaque, resulting in elevated vulnerable index of atherosclerotic plaque.

**Conclusion:**

These findings implicate MPO-catalyzed oxidization of HDL may contribute to atherosclerotic plaque instability by inhibiting SMC proliferation and migration through MAPK-ERK pathway which was dependent on SR-BI.

**Electronic supplementary material:**

The online version of this article (doi:10.1186/s12944-016-0388-z) contains supplementary material, which is available to authorized users.

## Background

High density lipoprotein (HDL) has been considered a protective factor of coronary heart disease (CHD). However, recent clinical trials showed that HDL-elevating medications failed to improve clinical outcomes in some CHD patients [1, 2], suggesting that HDL’s function is more important than its quality, which may be compromised with certain modifications. HDL’s protective role is also dependent on scavenger receptor BI (SR-BI), the major receptor for HDL [3]. Myeloperoxidase (MPO)-catalyzed oxidation, one of modifications for HDL, which is closely linked to CHD [4, 5], could be further classified into nitration (generating NO_2_-oxHDL) and chlorination (generating Cl-oxHDL). Proteomic studies have identified MPO as a component of advanced human atherosclerotic lesions, and revealed that HDL, more specifically, apolipoprotein A-I (apoA-I), the primary protein constituent of HDL, is a selective target for MPO-catalyzed nitration and chlorination in human serum and atherosclerotic lesions [6, 7]. NO_2_-Tyr (MPO-catalyzed nitration product) and Cl-Tyr (MPO-catalyzed chlorination product) contents are dramatically (6-fold higher than plasma) and selectively enriched within apoA-I recovered from atherosclerotic lesions in CHD patients [4, 8], oxidized apoA-I is in low abundance within the circulation, but accounts for 20% of the apoA-I in atherosclerotic plaque [9]. These results suggest an important role of MPO-catalyzed HDL nitration and chlorination in the pathogenesis of CHD.

Pathological studies showed that MPO expression was significantly increased only when atherosclerosis progresses into atherosclerotic plaque [5], and reached its highest level just before atherosclerotic plaque rupture [10]. Therefore, MPO has been considered a marker of unstable atherosclerotic plaque pathologically, angiographically and clinically [11–14]. Clinical studies also found that MPO serum levels could be used to predict the incidence of major adverse cardiac events (MACE) in patients with acute coronary syndrome (ACS) [15, 16]. These evidences identify MPO as a marker for unstable advanced plaque. Recently, MPO was found to form a complex with apoA-I and oxidize apoA-I in human atherosclerotic plaque of ACS patients [17], and serum concentrations of MPO also correlated with HDL serum levels in ACS patients [18]. Moreover, apoA-I was also found over-abundant in advanced plaques from unstable angina pectoris patients and co-localized with MPO [19], which raised an interesting question-whether MPO weakens plaque stability in advanced atherosclerotic lesions through oxidation of HDL. Pathological studies revealed significantly reduced vascular smooth muscle cells (SMCs) in unstable atherosclerotic plaques [20], and it is well known that plaque stability in advanced atherosclerotic lesions is increased by proliferation and migration of SMCs, but decreased by SMC apoptosis [21–23]. It was previously reported that HDL stimulates proliferation of SMCs [24, 25], which may increase plaque stability and contribute to HDL protection in CHD. Therefore, it is critical to investigate whether MPO-oxidized HDL has any adverse effects on SMCs, leading to plaque instability. Thus in this article we asked how MPO-nitrated and -chlorinated HDL modulates SMC migration, proliferation and apoptosis, and its direct impact on atherosclerotic plaque stability.

## Methods

### Isolation of HDL

Fresh, fasting plasma was separated by centrifugation from peripheral blood obtained from healthy subjects as described previously (referred to as HDL) [26]. The study protocol was approved by the local ethics committee. The clinical characteristics of plasma donors were listed in Table 1. Briefly, HDL was isolated from fresh plasma by ultracentrifugation (*d* = 1.063 to 1.21 g/mL), dialyzed against 3 × 1 L of endotoxin-free phosphate-buffered saline (PBS) with 100 μM diethylenetriamine pentaacetic acid (DTPA, Sigma, USA), sterilized with 0.22 μm filter, and stored in sealed tubes at 4 °C in dark until use within 1 month. The concentrations of HDL used in the present study were based on apoA-I content of HDL.

**Table 1:** Clinical characteristics of donors

	Healthy donors average (*n* = 10)
Age, Year	24.2 ± 2.66
Sex, Male/Female	6/4
Triglyceride, mmol/L	1.06 ± 0.48
Total Cholesterol, mmol/L	4.20 ± 0.67
HDL-C, mmol/L	1.26 ± 0.28
LDL-C, mmol/L	2.26 ± 0.58

Data are presented as mean ± SD as indicated. *HDL-C* high density lipoprotein cholesterol, *LDL-C* low density lipoprotein cholesterol, The donors were generally healthy with no medical history of hyperlipidemia, cardiovascular disease or other atherosclerotic disease, diabetes or hypertension. The donors denied the history of smoking, alcohol consumption, or drug use

### Animals

Three to six month old C57BL/6 background wild type (WT) mice were used to isolate aortic vascular smooth muscle cells. SR-BI (scavenger receptor BI) −/− mice (C57BL/6 background) were obtained from Dr. George Liu’s laboratory (Peking University, Beijing, China) [27]. Eight-week-old male ApoE−/− mice were purchsed from Charles River Laboratories (Wilmington, Massachusetts). Genotyping by PCR was performed using genomic DNA extracted from tails. These mice were kept under standard animal room conditions (temperature 21 ± 1°C; humidity 55–60%) with food and water continuously available for one week before the experiment.

### Animal model of carotid atherosclerosis

All ApoE−/− mice mice (male, 8 weeks old) received a high-fat diet (0.5% cholesterol and 15% cocoa butter) until sacrifice. After four weeks of high-fat diet, a silica-gel constrictive collar (0.30 mm inner diameter, 0.50 mm outer diameter, and 2 mm long) was placed around right carotid artery in all mice as described [28]. Three days after surgery, 200 μl of PBS (Phosphate Buffered Saline), 200 μl of native HDL, Cl-HDL, or NO2-HDL at an apoA-I concentration of 500 μg/ml was injected via the tail vein every three days for 4 weeks. The mice were euthanized by overdose of anesthetics at the end of experiment.

### Plaque Morphology & Histochemical Analysis

Atherosclerotic plaques at the aortic valve level were sectioned. Plaque morphological characters were analyzed via hematoxylin/eosin (H&E) staining. Plaque composition was detected by oil red O staining (lipid-rich cores), Sirius staining (collagen), Masson staining (fibrin), anti–α smooth muscle actin antibody (smooth muscle cells) staining, and anti–CD68 antibodies (macrophages) staining. Slides were visualized under a bright-field microscope (Leica DM2500, Tokyo, Japan), and pictures of the entire slice were taken with identical exposure settings for all sections and analyzed using image analysis software (ImageJ, National Institutes of Health, Bethesda, MD, USA). The abundance of each component was evaluated as percent of the vascular intima area. The vulnerable index was calculated as follows: the relative positive staining areas of (macrophages% + lipid%)/the relative positive staining areas of (SMCs% + collagen%) [29].

### Statistical analysis

All experiments were performed in triplicate unless indicated. The results of multiple observations are presented as the means ± SEM or as a representative result. Data were analyzed with SPSS version 19.0 (IBM SPSS, Armonk, NY, USA). Kolmogorov-Smirnov test was employed to analyze native distribution, independent sample t-tests was used to compare the difference between groups, difference was considered significant when *p* < 0.05.

Other materials and methods please refer to Additional file 1.

## Results

### MPO oxidation results in Cl-oxHDL and NO_2_-oxHDL

**Fig. 1:**
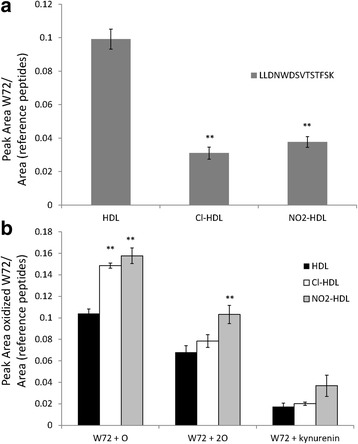
Mass Spectrometry analysis of HDL and MPO oxidized HDL. **a** Results from the selected ion monitoring (SIM) analysis of the native HDL, Cl-HDL and NO2-HDL samples. The data were obtained by normalizing the peak areas for the W72 containing peptides (LLDNWDSVTSTFSK) by the peak areas of the reference peptides. The reference peptides utilized in these experiments are the unmodified apoA-I peptides ATEHLSTLSEK and QGLLPVLESFK. **b** Oxidized W72 of the native HDL, Cl-HDL and NO2-HDL samples was also analyzed, with singly oxidized product, W + O (16 Da), doubly oxidized product, W + 2O (32 Da), and the oxidation of W to kynurenine (W + 4 Da) (**p < 0.01 compared with HDL group)

We have found previously through Mass Spectrometry that major protein component of HDL, apoA-I, is specifically modified upon MPO-catalyzed nitration and chlorination [26]. We showed that the tyrosine (Y) chlorination and nitration events were identified only in the MPO-treated samples. The chlorinated Y peptide was identified only in Cl-HDL and the nitrated Y peptides were identified only in NO_2_-HDL samples [26], which proved the specificity of our method. Importantly, it was found recently that a 2-oxindolyl alanine (2-OH-Trp) moiety at Tryptophan (W) 72 of apoA1 is critical in MPO-mediated inhibition of the ATP-binding cassette transporter A1 (ABCA1)-dependent cholesterol acceptor activity of apoA1 in vitro and in vivo [9]. ApoA1 containing a 2-OH-W72 group is abundant in atherosclerosis-laden arteries and could predict CHD risk [9]. Here we found that MPO-catalyzed nitration and chlorination also results in the oxidation of W72 to form the singly oxidized product, W + O (16 Da), doubly oxidized product, W + 2O (32 Da), and the oxidation of W to kynurenine [30] (W + 4 Da) (Fig. 1). These findings reflected the pathophysiological importance of the MPO oxidized HDL in our experiments.

### Cl-oxHDL and NO_2_-oxHDL have reduced capacity to stimulate SMC proliferation

**Fig. 2:**
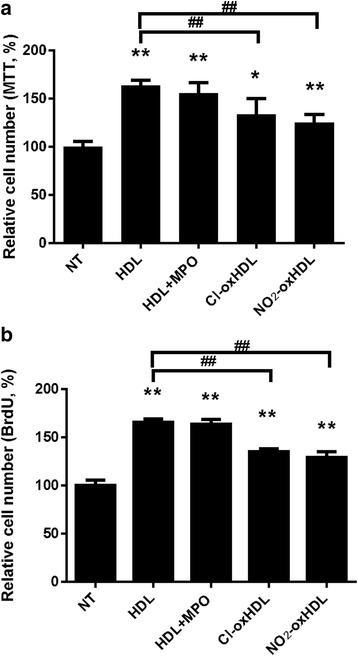
oxHDL reduces HDL’s promotion of SMC proliferation. **a** In vitro MTT assay was performed to analyze SMC viability. The native HDL and oxHDL was applied to SMCs at a concentration of 100 μg/ml for 48 h. The absorbance at A570 was measured and compared. **b** In vitro BrdU assay was performed to analyze SMC proliferation. The native HDL and oxHDL were applied to SMCs at a concentration of 100 μg /ml for 48 h. The absorbance at A450 was measured and compared (*p < 0.05, **p < 0.01 compared with non-treated group (NT), ##*p* < 0.01 compared with HDL group)

In order to examine the effects of Cl-oxHDL and NO_2_-oxHDL on SMC proliferation, we employed MTT and BrdU assays. We found that after 48 h treatment with 100 μg /ml native HDL, cell number increased by about 50% compared with non-treated cells as indicated by the MTT assay (Fig. 2a). To exclude the effect of MPO on cell proliferation, we also set up MPO + HDL group in the MTT assay, but the results showed no significant difference between MPO + HDL and HDL groups. However, Cl-oxHDL and NO_2_-oxHDL had significantly diminished stimulatory effects on cell proliferation compared with native HDL (Fig. 2a), with their MTT values 33% and 24% higher than the non-treated group, respectively. As shown in Fig. 2b, BrdU assay showed similar results. DNA synthesis was stimulated by about 60% in cells treated with HDL or HDL + MPO compared with cells in non-treated group. Cl-oxHDL and NO_2_-oxHDL groups were associated with reduced stimulation of DNA synthesis compared with native HDL (*p* < 0.01), but still stimulated DNA synthesis by 35% and 29%, respectively, compared with non-treated group. Results in Additional file 1: Figure S1B revealed that the stimulatory effects on BrdU incorporation were time-dependent.

### Cl-oxHDL and NO_2_-oxHDL inhibited SMC migration

**Fig. 3:**
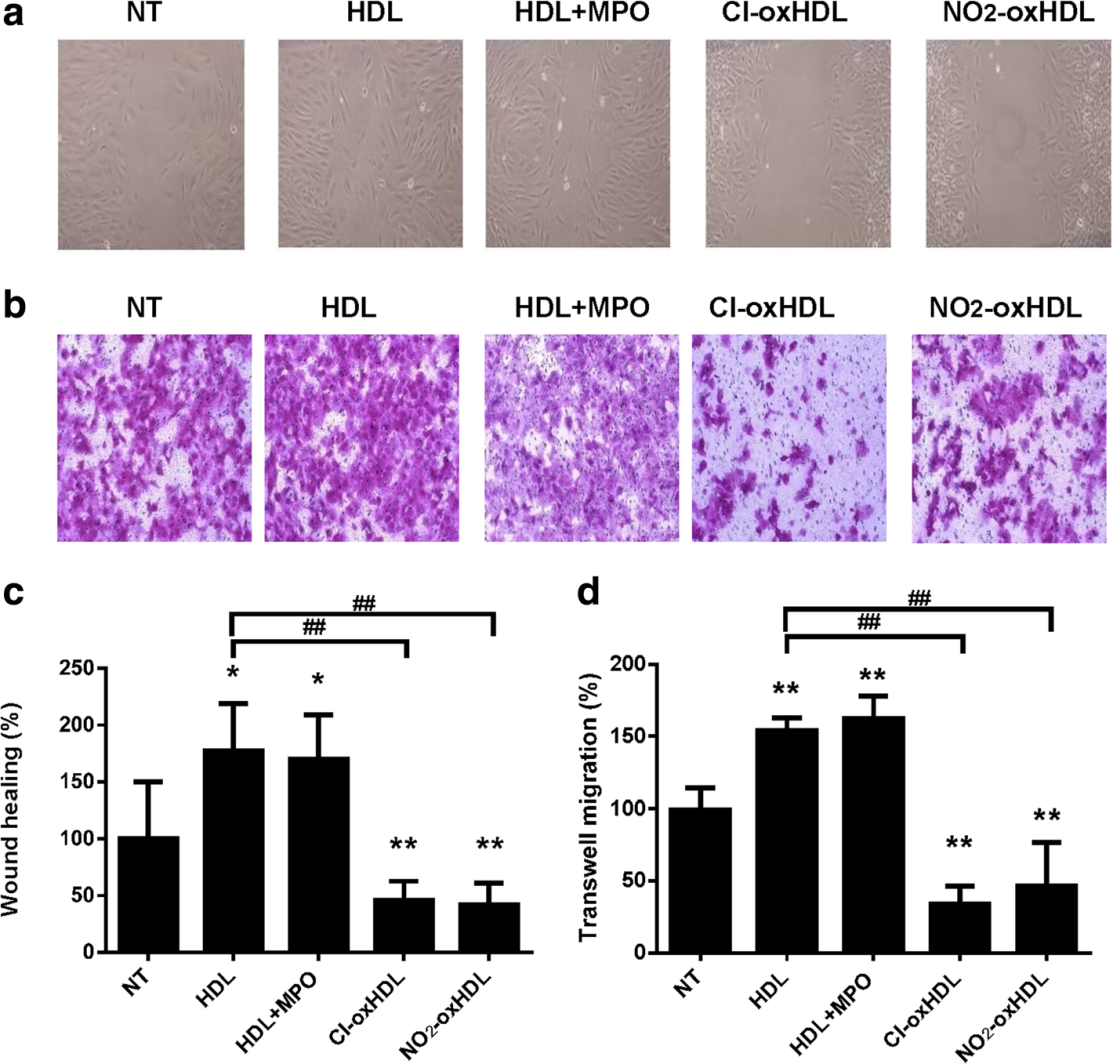
Oxidation of HDL inhibits SMC migration while HDL promotes SMC migration. **a** Primary aortic SMCs were cultured on 12-well plate until confluent, when an in vitro wound healing assay was performed. The native HDL and oxHDL were applied to SMCs at a concentration of 100 μg /ml for 48 h. Photographs of cells were taken 48 h after scratch. **b** In vitro transwell assay was performed to analyze SMC migration. The native and oxHDL were applied to SMCs at a concentration of 100 μg /ml for 12 h. The cells were stained and photographed. **c** The cells in wound healing assay were photographed 0 and 48 h after scratch, the relative area of SMC migration was compared. **d** Migrated cells in transwell assay were counted and compared (*p < 0.05, **p < 0.01 compared with non-treated group (NT), ##*p* < 0.01 compared with HDL group)

We first used in vitro wound healing assay to examine Cl-oxHDL and NO_2_-oxHDL’s effects on SMC migration. We found that HDL increased the migration of SMCs by 70% compared with non-treated cells. However, Cl-oxHDL and NO_2_-oxHDL inhibited SMC migration by about 54% and 58%, respectively, compared with non-treated cells (Fig. 3a and c). We also used transwell assay to confirm these findings. As shown in Fig. 3b and d, HDL increased SMCs migration by about 50% compared with non-treated cells, while Cl-oxHDL and NO_2_-oxHDL inhibited SMC migration by about 60% and 50%, respectively, compared with non-treated cells. Data in Additional file 1: Figure S1A confirmed that the transwell migration was in dose-dependent manner. These results clearly showed that HDL of healthy subjects promotes the migration of SMCs, while MPO catalyzed oxHDL inhibits SMC migration.

### Cl-oxHDL and NO_2_-oxHDL had no significant effects on SMC apoptosis

We used Annexin-V apoptosis assay to examine Cl-oxHDL and NO_2_-oxHDL’s effects on SMC apoptosis. We found that 24 h treatment with either 100 μg /ml native or oxHDL did not significantly affect SMC apoptosis (Additional file 1: Figure S2).

### Cl-oxHDL and NO_2_-oxHDL have diminished capacity to activate ERK1/2 phosphorylation

**Fig. 4:**
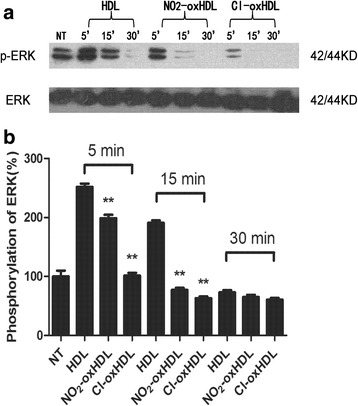
HDL stimulates ERK pathway while oxHDL inhibits such pathway. **a** The native and oxHDL were applied to SMCs at a concentration of 100 μg /ml for 5, 15 and 30 min, before cells were harvested for western blot. **b** The band density of phospha-ERK (P-ERK), ERK was determined by Photoshop 7.0 software. The ratio of pERK/ERK were compared. (**p < 0.01 compared with HDL group)

The mitogen-activated protein kinase (MAPK)/ extracellular regulated protein kinases (ERK) pathways have been proved to play a vital role in modulating SMC migration and proliferation [25, 31]. The phosphorylation state of ERK1/2 is the marker for activation of MAPK/ERK pathway [32]. To further elucidate the mechanism by which Cl-oxHDL and NO_2_-oxHDL inhibit SMC migration, we determined whether ERK1/2 phosphorylation was affected. SMCs were treated with native HDL, Cl-oxHDL and NO_2_-oxHDL at an apoA-I concentration of 100 μg/ml for 5, 15, and 30 min. In Fig. 4a and b, our results showed that ERK1/2 phosphorylation in the three groups reached maximum at the same time point (5 min) and treatment with native HDL significantly increased the level of ERK1/2 phosphorylation by about 150%. However, Cl-oxHDL and NO_2_-oxHDL had dramatic reduction in stimulating phosphorylation of ERK1/2 compared with native HDL. Cl-oxHDL and NO_2_-oxHDL treatment for 5 min resulted in about 60% and 20% reduction of ERK1/2 phosphorylation, respectively, compared with HDL group. These results illustrate that Cl-oxHDL and NO_2_-oxHDL have diminished capacity to activate ERK1/2 as indicated by their phosphorylation.

### Cl-oxHDL and NO_2_-oxHDL inhibit SMC migration through the MAPK pathway

**Fig. 5:**
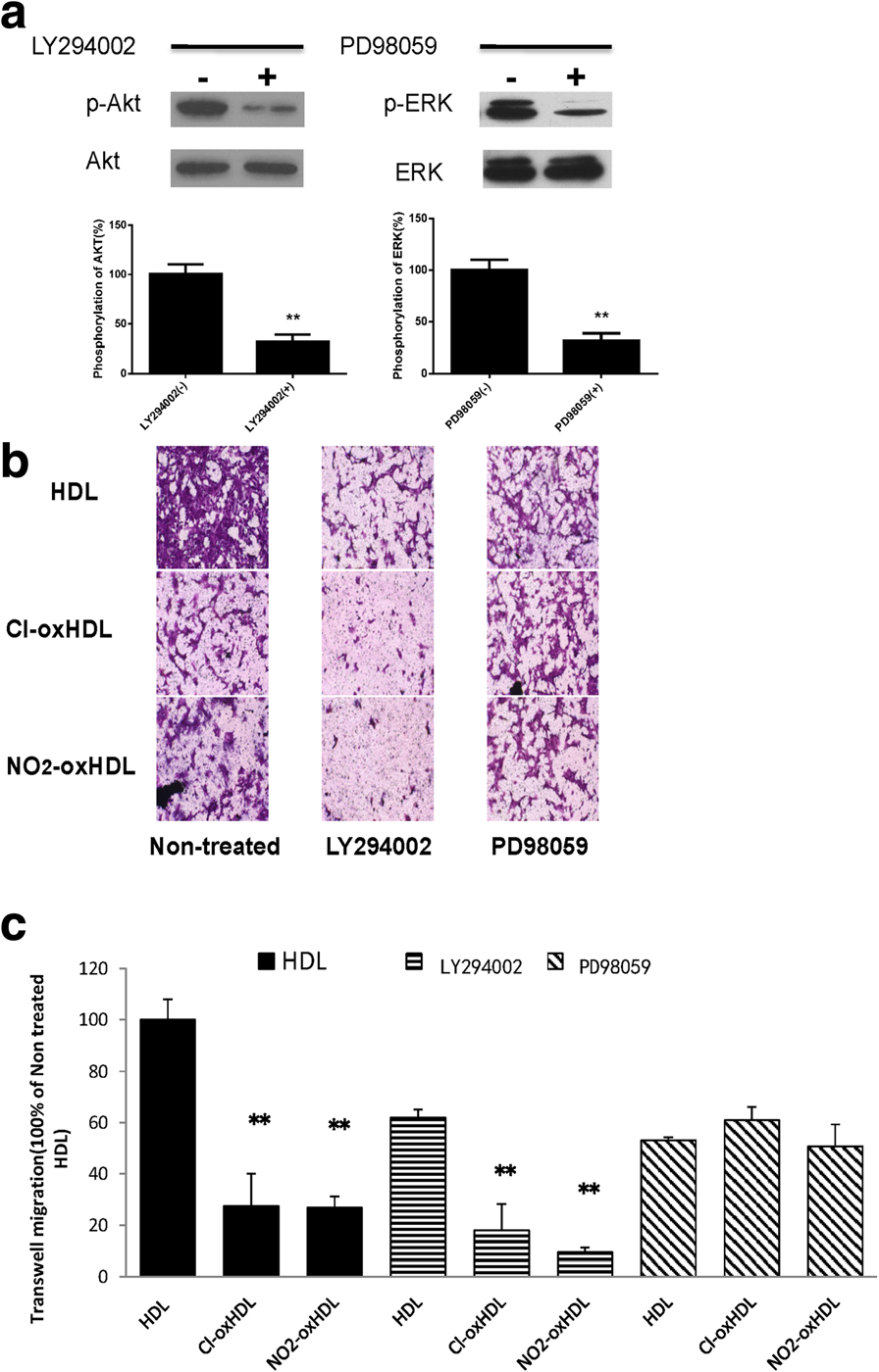
PD98059, but not LY294002, reverses inhibitory effects of oxHDL on SMC migraiton. **a** SMCs were preincubated with in the presence or absence of LY294002 (50 μM) or PD98059 (10 μM) for 12 h. Then cells were treated with HDL for 5 min, cell lysates were analyzed by western blot. **b** LY294002 and PD98059 were applied 12 h before transwell experiments were performed. The migrating cells were stained and photographed after 12 h incubation. **c** The number of migrated cells were counted and compared, normalized to the number of migrated cells in non-treated HDL group (**p < 0.01 compared with HDL groups in each treatments)

To test whether Cl-oxHDL and NO_2_-oxHDL inhibit SMC migration through MAPK pathway, we pre-treated SMCs with LY294002 (a PI3K inhibitor) and PD98059 (a MAPK inhibitor), and repeated the transwell assay. As shown in Fig. 5, PD98059 treatment abolished the differences of SMC migration responses between HDL, Cl-oxHDL and NO_2_-oxHDL groups, while LY294002 failed to do so. These results suggested that Cl-oxHDL and NO_2_-oxHDL inhibit SMC migration through MAPK but not PI3K/Akt pathway.

### HDL, Cl-oxHDL and NO_2_-oxHDL’s effects on SMC migration are dependent on SR-BI

**Fig. 6:**
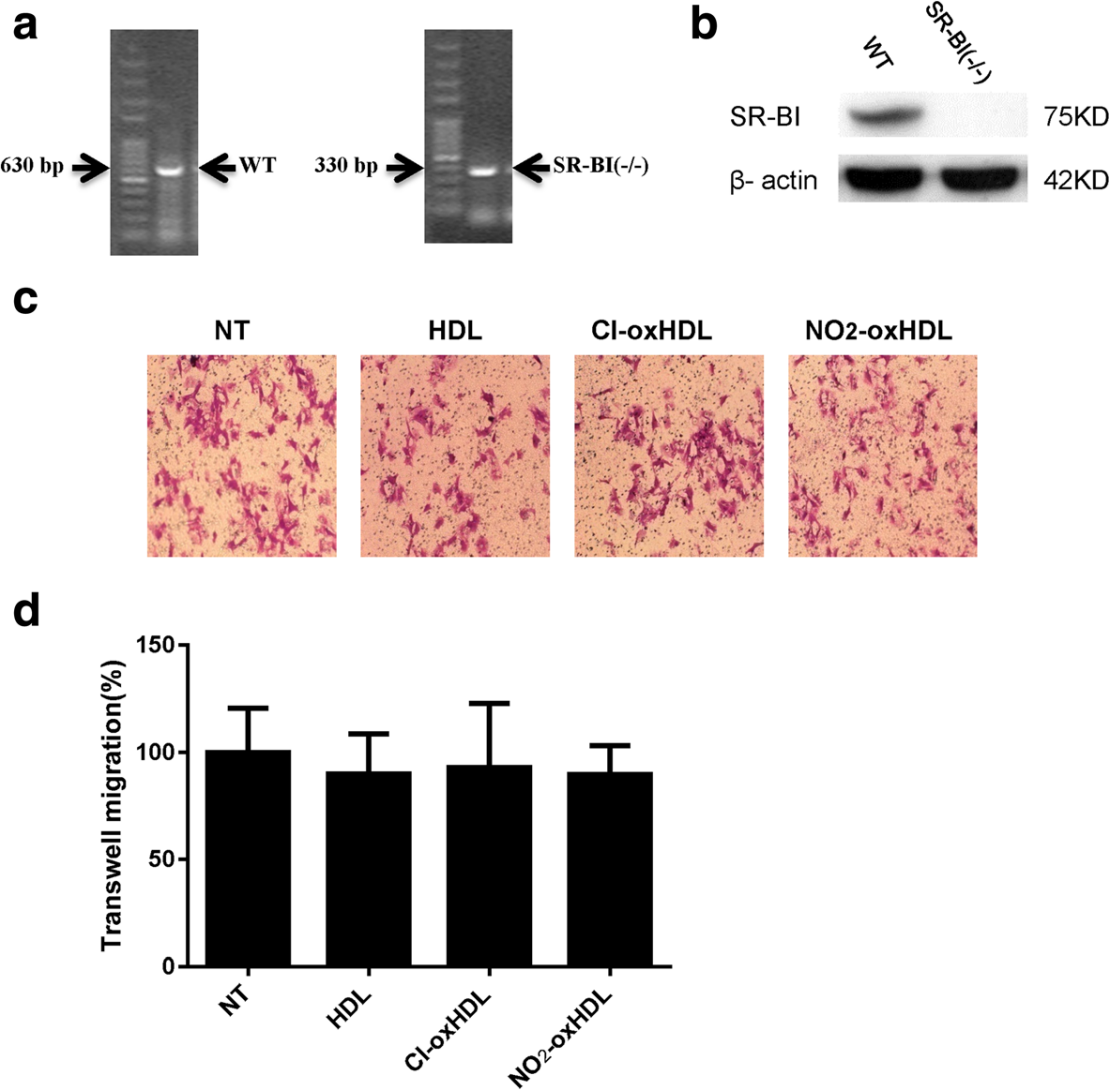
Native and oxHDL’s effects on SMC migration are dependent on SR-BI. **a** Representative agarose gel of SR-BI genotype assay by PCR. Wild type (WT) mice were identified by a band at 630 bp, while SR-BI (−/−) mice were identified by band at 330 bp. **b** Western blot analysis of SMCs from wild type (WT) and SR-BI (−/−) mice. **c** In vitro transwell assay was performed to analyze aortic SMC migration in SR-BI (−/−) mice. The native and oxHDL was applied to SMC at a concentration of 100 μg /ml for 12 h. The cells were stained and photographed. **d** Migrating cells were counted and compared (no significant difference was observed between the four groups)

HDL’s cholesterol transport function needs binding with its receptor Scavenger Receptor Class B Type I (SR-BI) [33]. It was reported recently that in endothelial cells and breast cancer cells, HDL modulates proliferation and migration through SR-BI [27, 34]. We reported previously oxidized HDL can inhibit SR-BI expression in endothelial cell [26]. However, it remains unknown in SMCs, whether HDL modulates migration through SR-BI. To determine whether HDL, Cl-oxHDL and NO_2_-oxHDL’s effects on SMC migration were dependent on SR-BI, we performed transwell experiments using SMCs derived from SR-BI (−/−) mice. Representative agarose gel of SR-BI mouse genotype assay by PCR was shown in Fig. 6a. The lack of SR-BI expression in the SR-BI (−/−) SMCs was confirmed by western blot (Fig. 6b). As seen in Fig. 6c and d, there was no significant difference among migration responses of SR-BI (−/−) SMCs treated without (non-treated) and with native HDL or oxHDL. These results indicated that native and oxHDL’s effects on SMC migration may be dependent on SR-BI.

### Cl-oxHDL and NO_2_-oxHDL inhibit platelet-derived growth factor (PDGF)-induced migration of SMCs

In atherosclerosis, PDGF-BB released from platelets, monocytes and endothelial cells could significantly promote SMC migration [35]. Reports have shown that HDL could inhibit PDGF-BB-stimulated SMC migration [36, 37]. Therefore, to answer whether Cl-oxHDL and NO_2_-oxHDL could influence PDGF-BB-stimulated SMC migration, we performed transwell assay in the presence of 30 ng/ml PDGF-BB. As shown in Additional file 1: Figure S3, native HDL significantly suppressed PDGF-BB stimulated SMC migration, while Cl-oxHDL and NO2-oxHDL could still significantly suppress SMC migration compared with native HDL.

### HDL, Cl-oxHDL and NO_2_-oxHDL’s effects on atherosclerotic plaque stability

**Fig. 7:**
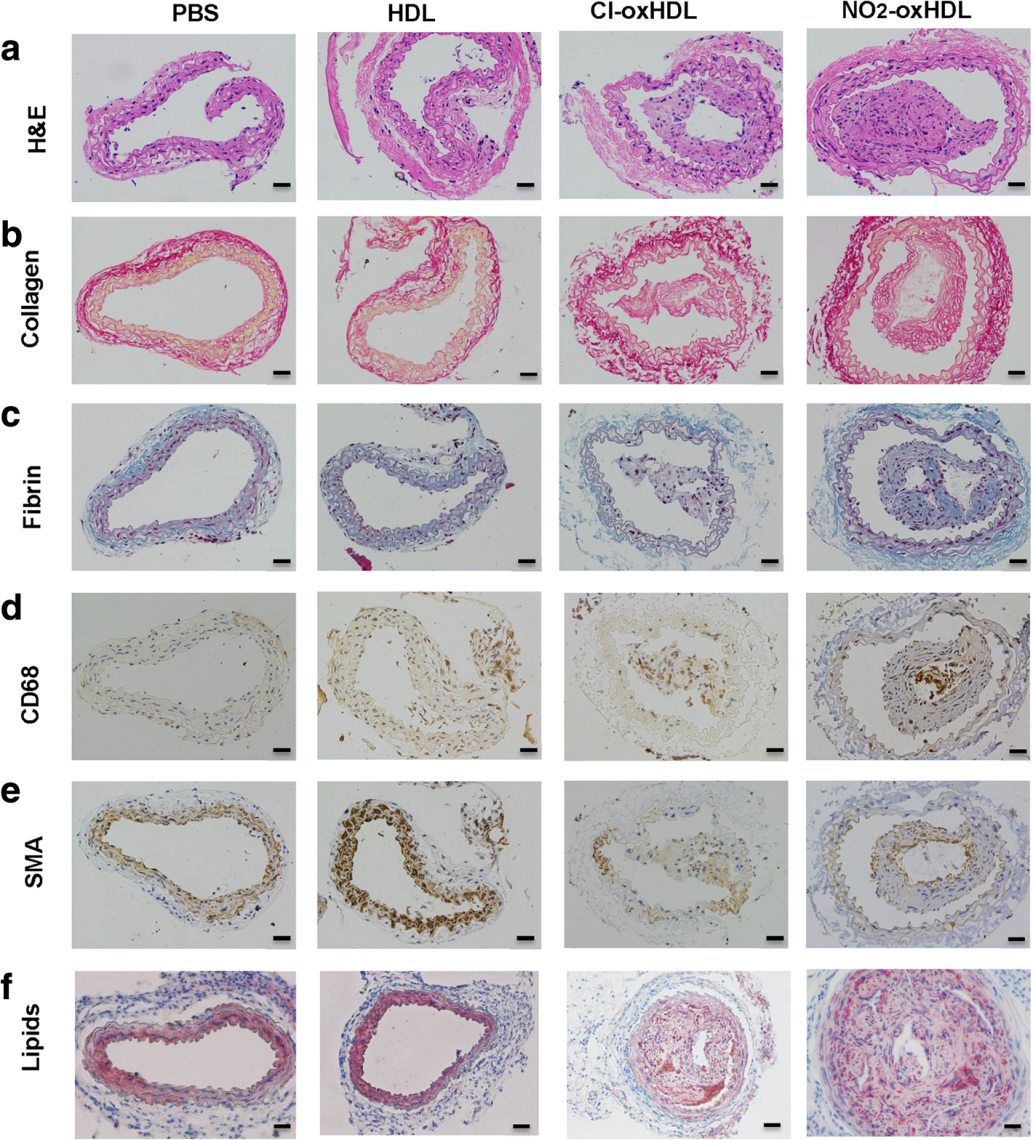
Native and oxHDL’s effects on plaque stability. **a** Representative images of H&E staining; **b** Representative images of Sirius red staining; **c** Representative images of Masson staining; **d** Representative images of immunostaining for CD68; **e** Representative images of immunostaining for α-SMC actin; **f** Representative images of Oil Red O staining (*n* = 6 in each group, scale bar = 50 μm)

**Fig. 8:**
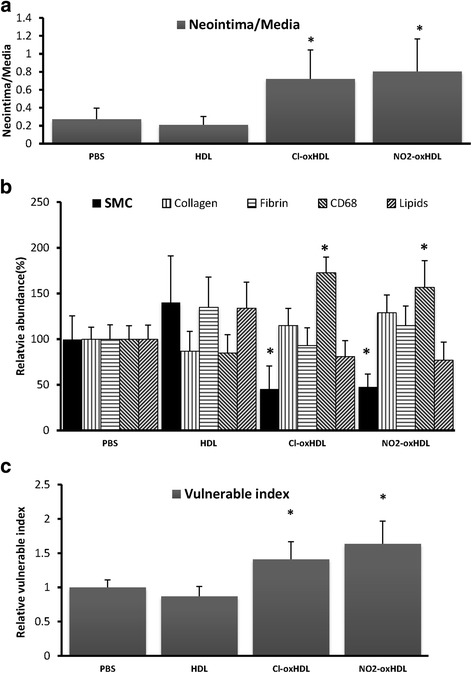
Native and oxHDL’s effects on plaque morphology & stability. **a** Statistical results of neointima/media ratio; **b** Statistical results of relative abundances (The abundance of each component was evaluated as percent of the vascular intima area) of SMA, collagen, fibrin, CD68 and lipids; **c.** Statistical results of plaque vulnerable index (values are mean ± SEM, *p < 0.05 compared with HDL group)

To determine the effects of HDL, Cl-oxHDL and NO_2_-oxHDL’s on atherosclerotic plaque stability, we performed coratid artery collar implantation in apoE−/− mice on high-fat diet. Same amount of PBS, HDL, Cl-oxHDL and NO_2_-oxHDL (200 μl, at the concentration of 500 μg/ml) was injected via tail vein every three days after collar implantation. At 4 weeks after collar implantation, we found increased neointima to media ratio in Cl-oxHDL and NO_2_-oxHDL treated groups compared with HDL group (*p* < 0.05, Figs. 7 and 8a). Moreover, significantly less SMC positive staining cells and significantly more CD68 positive staining cells were present in Cl-oxHDL and NO_2_-oxHDL treated groups compared with HDL group (*p* < 0.05, Figs. 7 and 8b), while no significant difference of collagen, fibrin and lipids staining was noticed between the four groups (Figs. 7 and 8b). We calculated the vulnerable index as described [29], and found significantly elevated relative vulnerable index in Cl-oxHDL and NO_2_-oxHDL treated groups compared with HDL group (*p* < 0.05, Fig. 8c).

## Discussion

In the current study, we have examined whether MPO-oxidized HDL (NO_2_- and Cl-oxHDL) modulates SMC proliferation, migration and apoptosis. Our results have revealed: 1) NO_2_-oxHDL and Cl-oxHDL inhibit SMC migration through the MAPK-ERK pathway dependent of SR-BI, 2) NO_2_-oxHDL and Cl-oxHDL inhibit PDGF-induced SMC migration, 3) NO_2_-oxHDL and Cl-oxHDL have impaired capacity to stimulate proliferation of SMCs, 4) NO_2_-oxHDL and Cl-oxHDL did not increase apoptosis of SMCs, and 5) NO_2_-oxHDL and Cl-oxHDL induced neointima formation and reduced SMC positive staining cells in atherosclerotic plaque.

At the early stage of atherosclerotic development, migration and proliferation of vascular SMCs play critical roles in neointima formation [35]. At the late stage, apoptosis of SMCs reduces plaque stability in atherosclerotic lesions, but migration and proliferation of SMCs enhance stability of atherosclerotic plaque [21–23]. HDL, a well-accepted cardioprotective factor, was reported to promote SMC proliferation [24, 25, 38], likely contributing to its cardioprotection [1, 39]. Notably, previous studies have clearly demonstrated a positive correlation between serum MPO and acute coronary syndrome in patients [17, 18], strongly suggesting a role of MPO in instability of atherosclerotic plaque. Although our results showed that MPO-oxidized HDL did not increase apoptosis, we have demonstrated that both NO_2_-oxHDL and Cl-oxHDL oxidized by MPO significantly inhibit SMC migration and lose their capacity to stimulate SMC proliferation compared with native HDL. It remains unaddressed here whether MPO-oxidized HDL inhibits proliferation of SMCs induced by other mitogens. However, we have demonstrated that HDL inhibits PDGF-induced migration of vascular SMCs, which is consistent with previous studies [36, 37]. Notably, MPO-oxidized HDLs are even more potently in inhibition of PDGF-induced migration of SMCs.

In an animal model of carotid atherosclerosis, we found NO_2_-oxHDL and Cl-oxHDL induced neointima formation while reduced SMC positive staining cells in atherosclerotic plaque, resulting in elevated vulnerable index of atherosclerotic plaque. Previous studies showed injection of recombinant apoA1 could reduce plaque burden [40, 41]. While in our experiments, we used native human HDL injection in a collar implantation model, which may explain the differences of results. Taken together, our data have provided at least partial explanation to the mechanism underlying MPO-related plaque instability [11–14].

Although we have not studied further why MPO-oxidized HDLs have impaired capacity to stimulate proliferation of SMCs compared with native HDL, we have investigated the signaling mechanisms underlying inhibition of SMC migration by oxHDLs. Our results have shown that HDL stimulates migration of SMCs, to our surprise, MPO-oxidized HDL even reduced the basal level of migration activities. It appears that either HDL-induced stimulation or oxHDL-induced inhibition of migration of SMCs requires the presence of SR-BI, because neither HDL nor NO_2_-oxHDL and Cl-oxHDL induced any changes in migration responses in SMCs derived from SR-BI (−/−) mice. Whether NO_2_-oxHDL and Cl-oxHDL can inhibit SR-BI expression or MPO-oxidized HDL lower the binding between HDL and SR-BI needs further study. The opposite effects on migration response by HDL and NO_2_-oxHDL and Cl-oxHDL may result from differential effects on the MAPK pathway. First, previous studies have shown that activation of the MAPK pathway plays a critical role in migration of vascular SMCs. Indeed, treatment with PD98059 significantly inhibited HDL-induced migration responses. Second, NO_2_-oxHDL and Cl-oxHDL induced only a transient activation of MAPK. In response to treatment with NO_2_-oxHDL, phosphorylation of MAPK reduced to the basal level at 5 min, which were almost undetectable at 15 min. In response to treatment with Cl-oxHDL, the phosphorylation level of MAPK at 5 min was much lower than the basal level. Finally, PD98059 treatment could reverse the inhibition of migration response of SMCs induced by both NO_2_-oxHDL and Cl-oxHDL.

**Fig. 9:**
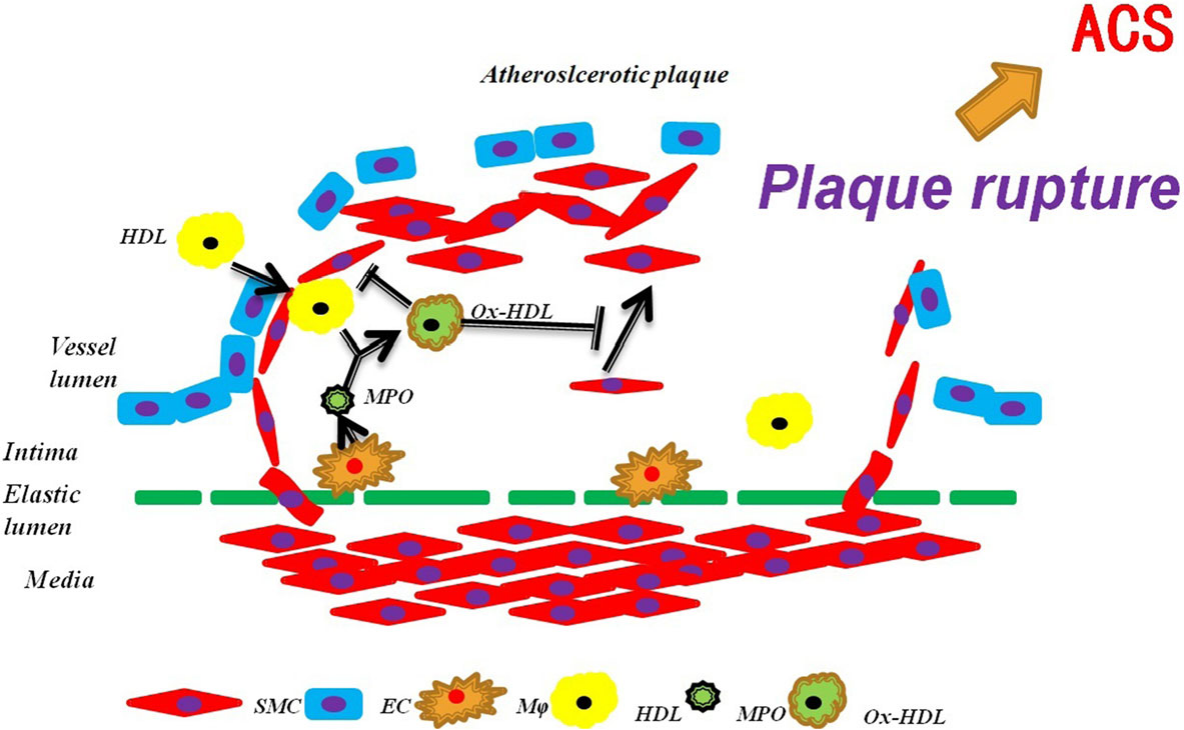
Cartoon of a hypothesis on MPO’s role in plaque stability. SMC = smooth muscle cell; EC = endothelial cell; Mφ = macrophage; HDL = high density lipoprotein; MPO = myeloperoxidase; Ox-HDL = MPO oxidized HDL

Given that MPO is a hallmark of unstable atherosclerotic plaque and acute coronary events [11–14] and recent reports show that HDL is associated with MPO in ACS patients [17–19], our findings demonstrating that MPO-catalyzed HDL nitration and chlorination regulate SMC proliferation and migration have proposed a novel hypothesis on MPO’s role in plaque stability (Fig. 9). In atherosclerotic plaque, HDL was oxidized by MPO released from macrophages and generated Cl-oxHDL and NO_2_-oxHDL, which inhibited SMC migration and proliferation, resulting in reduced SMC population in the fibrous cap, which may ultimately lead to plaque instability.

## Conclusion

Our findings implicate MPO-catalyzed oxidization of HDL may contribute to atherosclerotic plaque instability by inhibiting SMC proliferation and migration through MAPK-ERK pathway which was dependant on SR-BI, which may surve as potential therapeutic target in ACS.

## Additional file

Additional file 1: Supplemental methods and supplemental results. (DOC 125 kb)

## Abbreviations

ABCA1: ATP-binding cassette transporter A1
ACS: Acute coronary syndrome
apoA-I: Apolipoprotein A-I
CHD: Coronary heart disease
Cl-oxHDL: Chlorinated oxidized high density lipoprotein
ERK: Extracellular regulated protein kinases
HDL: High density lipoprotein
MACE: Major adverse cardiac events
MAPK: Mitogen-activated protein kinase
MPO: Myeloperoxidase
NO_2_-oxHDL: Nitrated oxidized high density lipoprotein
NT: Non treated
PBS: Phosphate buffered saline
PDGF: Platelet-derived growth factor
SMCs: Vascular smooth muscle cells
SR-BI: Scavenger receptor BI
W: Tryptophan
WT: Wild type
Y: Tyrosine
