# Supplementary material for: Myeloperoxidase-oxidized high density lipoprotein impairs atherosclerotic plaque stability by inhibiting smooth muscle cell migration
Source: Lipids Health Dis. 2017 Jan 10;16:3. doi: 10.1186/s12944-016-0388-z (PMC5223295; doi:10.1186/s12944-016-0388-z)
Supplement: Additional file 1: — Supplemental methods and supplemental results. (DOC 125 kb) [file 12944_2016_388_MOESM1_ESM.doc]

**MATERIALS AND METHODS**

***Protein digestion and peptide analysis*** The protein bands were digested according to an in-gel digestion procedure. Briefly, the bands were cut from the gel and washed in 50% ethanol/5% acetic acid, alkylated with iodoacetamide and reduced with dithiothreitol (DTT). The digestions were carried out with either a modified, sequencing-grade trypsin (Promega, Madison, WI) or overnight at room temperature. The peptides were extracted from the gel, evaporated to dryness, and reconstituted in 1% acetic acid for analysis. The mass spectrometry experiments were performed on an LTQ linear ion trap mass spectrometer (Thermo Scientific, San Jose, CA) equipped with a nano-electrospray source. The source is operated at a flow rate of 250 nl/min at an operating voltage of ~2.5 kV. The proteolytic peptides were analyzed by reverse phase HPLC using an Eksigent nano-1D HPLC system (Dublin, CA) with a self-packed 9cm x 75 µm Phemomenex Jupiter C18 column. The peptides were eluted using a 2-70% acetonitrile/ 0.1% formic acid gradient. The digests were analyzed in a data-dependent manner as previously described . Initial protein modification analysis was carried out by searching the LC-MS/MS data specifically against the sequence of apoA-I using the program Sequest bundled into Proteome Discoverer 1.3 (Thermo Scientific, San Jose, CA). Positively identified modifications were required to have Sequest Xcorr scores greater than 2.0 for [M+2H]^+2^ ions and 2.5 for [M+3H]^+3^ ions and these spectra were also subjected to manual inspection. The quantitation of the modified peptides was carried out using the native reference peptide method . This method used a selected ion monitoring (SIM) experiment to acquire the full MS and MS/MS spectrum for each tryptic digest. The mass of the ions along with the chromatographic elution times is used to construct chromatograms used in the quantitative analysis. The peak areas for each peptide were determined and normalized to the peak areas of the reference peptides.

***Isolation of SMCs and primary cell culture*** Mouse aortic SMCs were isolated and cultured as previously described. Briefly, mouse aortic SMCs were isolated from thoracic aortas of WT and SR-BI -/- mice. After removal of endothelial, adventitia and connective tissue, arterial media were cut into 1 mm^2^ segments, which were then placed in culture dishes in 37℃incubator with 5% CO_2_ for 2-4 h. Cells were cultured in DMEM (Hyclone) with 10% fetal bovine serum (FBS), 2 mM L-glutamine, 100 U/ml penicillin, and 100 μg/ml streptomycin. The medium was changed every 2 d, and cells were passaged by treatment with 0.2% trypsin and 0.02% EDTA solution. Cells up to passage 6 were used for the experiments.

***MTT cell proliferation assay*** Cell proliferation was evaluated by MTT assay. SMCs were plated on 96-well plate at a density of 4000 cells/well and cultured overnight, serum-starved in DMEM containing 0.5% FBS for 12 h, then cultured with medium containing 1% FBS alone or with 100 μg/ml of HDL, HDL+MPO, Cl-oxHDL or NO_2_-oxHDL, respectively, for 48 h. 4 h before the end of the incubation, 10 μl of 5 mg/ml 3-(4, 5-Dimethylthiazol-2-yl)-2, 5-diphenyltetrazolium bromide (MTT) (Ameresco, USA) was added to each well. After 4 h incubation with MTT, 200 μl/well DMSO (dimethylsulfoxide) was added after discarding MTT. The optical density of the solubilized formazan was read at 570 nm with an ELISA (Enzyme-Linked Immunosorbent Assay) plate reader (BioRad, USA). The average values were determined from quadruplicate readings.

***BrdU (5’-bromo-2’-deoxyuridine) incorporation assay*** The experiment was carried out using Roche Cell Proliferation Elisa BrdU kit (Roche 11647229001) according to the manufacturer’s instruction. SMCs were plated at a density of 4000 cells/well in 96-well plates and cultured overnight, serum-starved in DMEM containing 0.5% FBS for 12 h, followed by incubation with and without 100 μg/ml of HDL, HDL+MPO, Cl-oxHDL or NO_2_-oxHDL, respectively, for 48 h. Subsequently, the cells were labeled with 20 μl/well of BrdU labeling solution (10mM), and then incubated with 200 μl/well of FixDenat. After incubation with 100 μl/well of Anti-BrdU-POD working solution for 90 min, the cells were washed 3 times with wash buffer, followed by addition of substrate solution (TMB). The absorbance of each well was read at 450 nm with an ELISA plate reader (BioRad, USA).

***Wound healing assay*** SMCs were plated with DMEM containing 10% FBS in 12-well plate (5×10^5^ cells/well) and cultured till formation of cell monolayers, followed by serum starvation with DMEM containing 0.5% FBS for 12 h. Cell monolayers were then wounded by scratching with a 20 μl-micropipette tip, followed by washing 3 times with PBS. Cells were then incubated with DMEM containing 1% FBS alone or together with HDL, HDL+MPO, Cl-oxHDL or NO_2_-oxHDL, respectively, at 100 μg/ml for 48 h. Micrographs were taken using an inverted microscope (Nikon, Japan) at 0 and 48 h after scratch. The areas without cells were compared at 0 h and 48 h. The relative area of SMC migration was calculated as the difference between 0 h and 48 h. Results were confirmed in three independent experiments.

***Transwell migration*** SMC migration was also determined using a modified Boyden chamber (Millipore, USA) with 8.0-μm pore polycarbonate filter inserted in the 24-well plate. The lower chamber was filled with 600 μl of DMEM with 10% FBS. SMCs (10^5^ cells/well) in 0.1% bovine serum albumin DMEM with/without 100 μg/ml HDL, HDL+MPO, Cl-oxHDL or NO_2_-oxHDL were plated into the upper chamber, followed by incubation for 12 h. All non-migrating cells were removed from the upper face of the transwell membrane with a cotton swab, but migrated cells were fixed and stained with 0.1% crystal violet. Migrated cells were photographed in ten high power fields for each chamber.

For the studies with signal pathway inhibitors, SMCs were pre-incubated with and without LY294002 (50 μM) or PD98059 (10 μM ) for 12 h, followed by treatment with HDL, HDL+MPO, Cl-oxHDL or NO_2_-oxHDL at 100 μg/ml apoA-I in the upper chamber for 12 h before the photos were taken.

For the studies with PDGF-BB, 30 ng/ml PDGF-BB was added into the upper chamber. Cells were cultured for 8 h before the photos were taken.

***Detection of apoptosis*** Apoptotic cell death in SMCs was assessed by flow cytometry (Annexin V-EGFP Kit, Keygen KGA101). SMCs were plated at a density of 10^6^ cells/well in 6-well plates and cultured overnight, serum-starved in DMEM containing 0.5% FBS for 12 h and then incubated with and without 100 μg/ml of HDL, HDL+MPO, Cl-oxHDL or NO_2_-oxHDL, respectively, for 24 h. The cells were suspended by a brief trypsinization (0.05% without EDTA) and washed twice with cold PBS. Cells were then resuspended in 500 μL of 1x binding buffer with 5 μl annexin V-EGFP and 5 μl propidium iodide (PI). Cells were gently mixed and incubated for 15 min at room temperature in the dark. Fluorescence was induced with the 488-nm argon laser and monitored at 512 nm (FL1) for the FITC signal and 620 nm (FL2) for PI fluorescence on FACS Vantage. Apoptotic cells were identified as FITC and PI double positive. The percentage of apoptotic cells were normalized to non treated cells.

***Immunoblotting*** Phosphorylation of ERK1/2 and total ERK1/2 were analyzed by western blot. SMCs were cultured in 6-well plates, starved for 12 h in 0.5% FBS-containing DMEM, and treated in DMEM with/without 100 μg/ml HDL, HDL+MPO, Cl-oxHDL or NO_2_-oxHDL for the indicated time. After treatment, cells were harvested and lysed in a mixture containing radio-immunoprecipitation assay (RIPA) buffer (50 mM Tris -pH 7.4, 150 mM NaCl, 1% NP-40, 0.5% sodium deoxycholate, 0.1% SDS, 0.1% EDTA, 1% Triton X100), in the presence of protease inhibitors and phosphatase inhibitors (Applygen Technologies Inc., China). Cell debris was removed by centrifugation (12000 rpm for 20 minutes). The protein concentration was determined by Coomassie Brilliant Blue method. Cell lysates were analyzed by western blot using specific primary antibodies. Cell lysates (50 μg protein per lane) were subjected to electrophoresis on 10% SDS-polyacrylamide gels (SDS-PAGE) and transferred onto nitrocellulose membranes (Pall Corporation, USA) according to standard procedures. The membranes were blocked for 1 h with 5% non-fat milk containing 0.05% Tween-20 in Tris buffered saline. Membranes were incubated with each primary antibody (1:500 ‒ 1:2000 dilution, antibodies against ERK1/2 was from Santa Cruz sc135900, phospha-ERK from EPITOMICS 1481-1, and SR-BI from EPITOMICS 1971-1) overnight at 4°C, followed by the appropriate HRP-conjugated secondary antibodies diluted according to the manufacturers’ protocol. Antibody binding was detected using the SuperSignal West Pico Kit (Pierce, USA) according to the manufacturer’s instructions. The images were analyzed and semi-quantified using Adobe Photoshop software 7.0.

**REFERENCES**

1. Pan B, Yu B, Ren H, Willard B, Pan L, et al. (2013) High-density lipoprotein nitration and chlorination catalyzed by myeloperoxidase impair its effect of promoting endothelial repair. Free Radic Biol Med 60C: 272-281.

2. Zheng L, Settle M, Brubaker G, Schmitt D, Hazen SL, et al. (2005) Localization of nitration and chlorination sites on apolipoprotein A-I catalyzed by myeloperoxidase in human atheroma and associated oxidative impairment in ABCA1-dependent cholesterol efflux from macrophages. J Biol Chem 280: 38-47.

3. SNE KM (2000) Protein Sequencing and Identification Using Tandem Mass Spectrometry. New York: John Wiley and Sons, Inc.

4. Ruse CI, Willard B, Jin JP, Haas T, Kinter M, et al. (2002) Quantitative dynamics of site-specific protein phosphorylation determined using liquid chromatography electrospray ionization mass spectrometry. Anal Chem 74: 1658-1664.

5. Willard BB, Ruse CI, Keightley JA, Bond M, Kinter M (2003) Site-specific quantitation of protein nitration using liquid chromatography/tandem mass spectrometry. Anal Chem 75: 2370-2376.

6. Zhou B, Margariti A, Zeng L, Habi O, Xiao Q, et al. (2011) Splicing of histone deacetylase 7 modulates smooth muscle cell proliferation and neointima formation through nuclear beta-catenin translocation. Arterioscler Thromb Vasc Biol 31: 2676-2684.

**
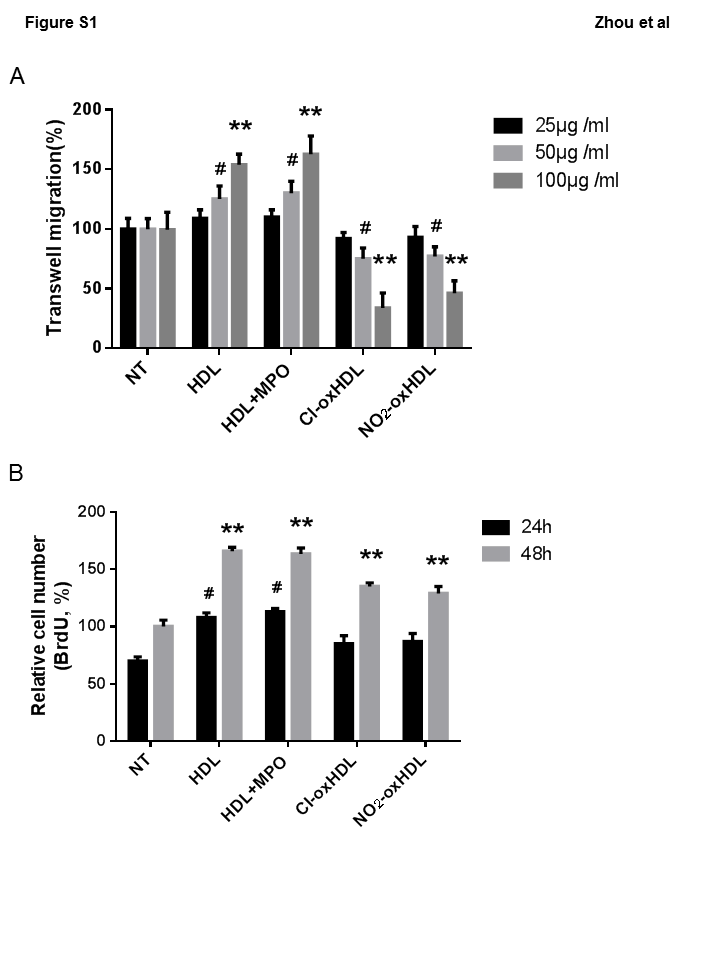
**

Figure S1. Oxidation of HDL inhibits SMC migration and proliferation in dose-dependent and time-dependent manners. A. In vitro transwell assay was performed to analyze SMC migration. The native and oxHDL were applied to SMCs at a concentration of 25, 50 and 100μg /ml for 12 h. Migration was compared with non-treated group (NT) (**p<0.01 compared with non-treated group at 100μg /ml, #p<0.05 compared with NT group at 50μg /ml). B. In vitro BrdU assay was performed to analyze SMC proliferation. The native HDL and oxHDL were applied to SMC at a concentration of 100μg /ml for 24 and 48 h(**p<0.01 compared with non-treated group for 48h, #p<0.05 compared with NT group for 24h).

**
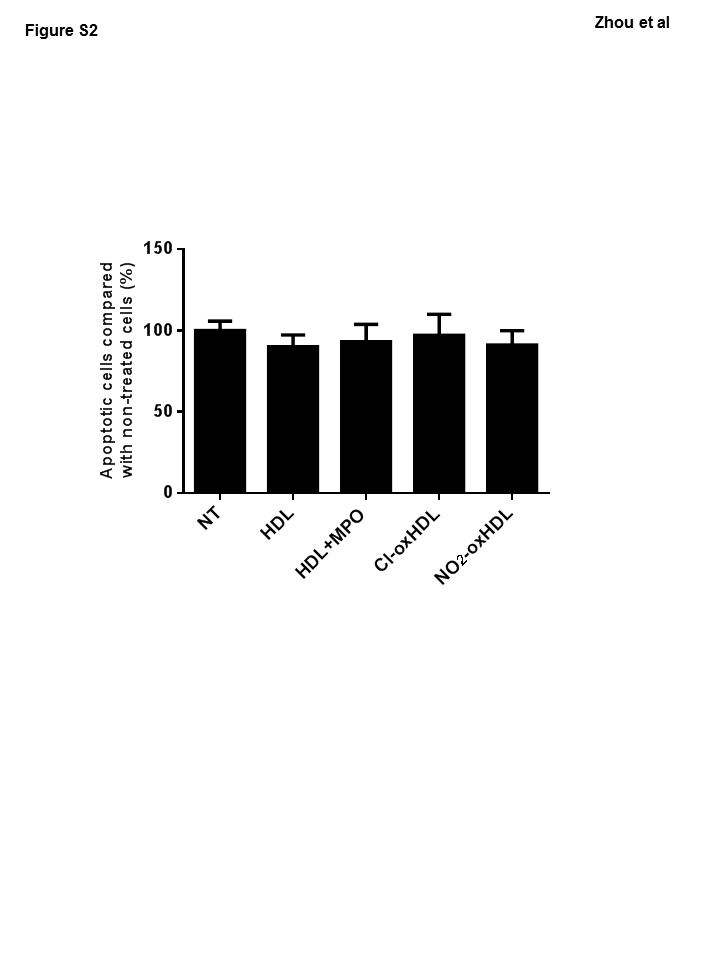
**

Figure S2. OxHDL has no significant influence on SMC apoptosis. *In vitro* Annexin V apoptosis assay was performed to analyze SMC apoptosis. The native HDL and oxHDL was applied to SMC at a concentration of 100μg /ml for 24 h. The cells were labeled with Annexin V-EGFP and PI, and apoptosis was assessed by flow cytometry. Annexin-V positive cells were compared with non-treated cells.

**
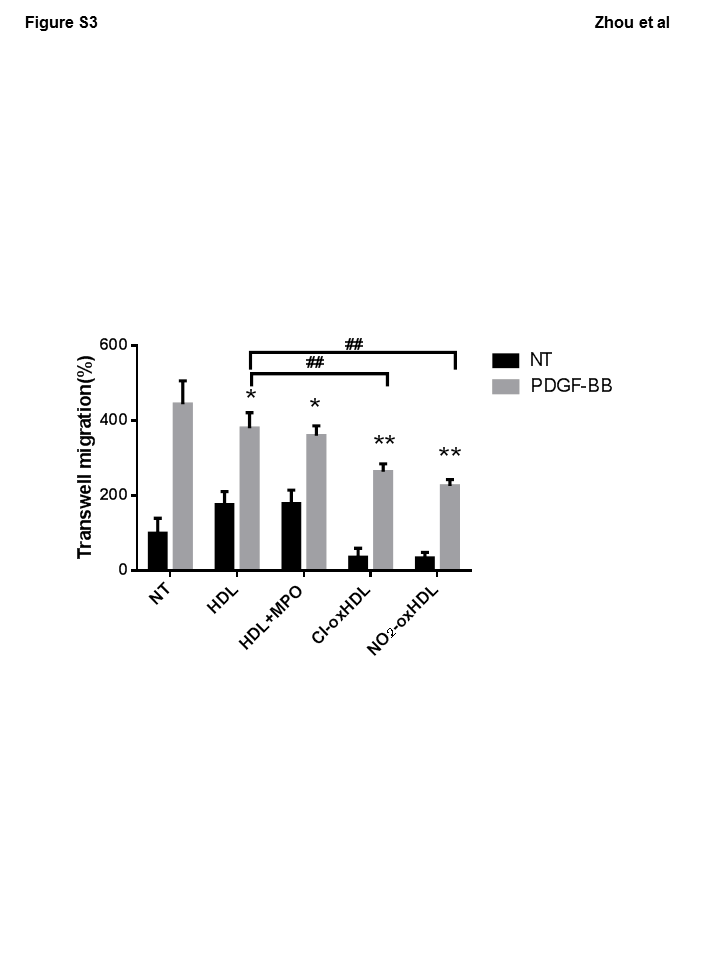
**

Figure S3. Oxidation of HDL inhibits PDGF-induced SMC migration. SMCs were treated with HDL and oxHDL at 100μg /ml in the presence or absence of 30 ng/ml PDGF-BB for 8 h. Migrating cells were stained and counted, normalized to the number of non-treated group (*p<0.05, **p<0.01 compared with PDGF-BB+NT group, ##p<0.01 compared with PDGF-BB+HDL group).
